# Multi-Threshold Remote Sensing Image Segmentation Based on Improved Black-Winged Kite Algorithm

**DOI:** 10.3390/biomimetics10050331

**Published:** 2025-05-19

**Authors:** Yi Zhang, Xinyu Liu, Wei Sun, Tianshu You, Xin Qi

**Affiliations:** 1 College of Electrical and Computer Science, Jilin Jianzhu University, Changchun 130119, China; zhangyi@jlju.edu.cn (Y.Z.); liuxinyu@student.jlju.edu.cn (X.L.); sunwei@jlju.edu.cn (W.S.); youtianshu@jlju.edu.cn (T.Y.); 2 Rural Revitalization Research Institute, Changchun Sci-Tech University, Changchun 130022, China

**Keywords:** multi-threshold image segmentation, remote sensing image, black-winged kite algorithm, remote sensing technology

## Abstract

This paper proposes an adaptive multi-threshold image segmentation method named IBKA-OTSU to address the limitations of existing deep learning-based image segmentation methods, particularly their heavy reliance on large-scale annotated datasets and high computational complexity. The proposed algorithm significantly enhances the capability of complex remote sensing scenarios by systematic improvements to core algorithm components, including population initialization strategy, attack behavior patterns, migration mechanisms, and opposition-based learning strategy. The improved intelligent optimization algorithm is innovatively integrated with the OTSU threshold method to establish a multi-threshold segmentation model specifically designed for remote sensing imagery. Experimental validation using representative samples from the ISPRS Potsdam benchmark dataset demonstrates that our IBKA-optimized OTSU multi-threshold segmentation method outperforms traditional IBKA-optimized pulse coupled neural network (PCNN) approaches in remote sensing image analysis. Quantitative evaluations reveal substantial improvements in the dice coefficient across six randomly selected remote sensing images, achieving performance enhancements of 7.76%, 11.99%, 30.75%, 22.91%, 44.37%, and 18.55%, respectively. This research provides an effective technical solution for intelligently interpreting remote sensing imagery in resource-constrained environments, demonstrating significant theoretical value and practical application potential in engineering implementations.

## 1. Introduction

Image segmentation is a critical aspect of image processing and has been a significant concern across various fields. The requirements for image processing vary depending on the type of image and its intended application. For example, remote sensing images are often derived from satellites and present challenges due to their large data volume, high complexity, and extensive size. Effective segmentation is essential to extract ground object information while ensuring strong robustness accurately. In the medical field, images may contain specific noise and artifacts. Accurately detecting lesions or tumors is crucial for subsequent diagnoses, which necessitates high-precision image segmentation. In autonomous driving, real-time processing of road information is vital. Consequently, image segmentation technology must adapt well to varying weather conditions, road types, and other environmental factors, imposing higher demands on its performance across different scenarios. Given the distinct characteristics and requirements of images in each field, selecting the appropriate segmentation technology is essential to meet specific objectives. This paper focuses on remote sensing image segmentation. Remote sensing technology has numerous applications, including meteorological observation, vegetation classification, and earthquake monitoring. The images processed in this context are often satellite images characterized by large sizes, complex backgrounds, and intricate details. Additionally, images may be acquired from different angles, leading to significant computational demands, extended processing times, and suboptimal segmentation results. Moreover, advancements in remote sensing technology have significantly enhanced the spatial resolution of images, allowing for more precise detail capture. As satellite imagery now covers a broader range of spectral bands, these developments bring forth additional challenges and requirements for effective remote sensing image processing.

Many remote sensing image segmentation methods have been gradually developed to cope with the above challenges. Current image segmentation technologies mainly include traditional methods such as threshold-based segmentation, edge detection-based methods, region segmentation, unsupervised clustering methods, graph theory [1], and methods based on deep learning. Edge detection technology can provide good detection accuracy for the original image. However, the direct use of the effect could be better [2] and generally needs to be combined with other methods to improve segmentation ability. The gray histogram of the image segments the threshold method. Because the threshold value needs to be set manually, it brings much inconvenience to the segmentation. The literature [3] proposes a method based on maximum inter-class variance (OTSU) to select thresholds automatically to solve this problem. Although this method performs well in simple scenes, it could have affected complex backgrounds and multi-level segmentation problems better. Therefore, many improved OTSU methods have been proposed. Literature [4,5] optimized the original OTSU method, but there are also problems, such as low accuracy and many noise points. Because of its robustness to noise and outliers, clustering image segmentation methods are often used in the field of image segmentation, such as the k-means clustering algorithm (K-means) [6,7] and fuzzy C-means clustering (FCM) [8,9]. In literature [6], the subtraction clustering method is used to generate the initial cluster center, and the given pixel points are repeatedly assigned to the corresponding center point according to the specified distance formula, which is applied to the k-means algorithm for image segmentation. Literature [9] combined elastic fuzzy C-means with a smoothing method to better use neighborhood information and improve the inclusion and accuracy of segmentation. Weighted multi-view K-means clustering with L2 regularization (W-MV-KM-L2) [10] based on weighted multi-view K-means clustering (W-MV-KM) is specially designed for multi-view data clustering. Assign different degrees of importance to each view. Unsupervised MV-FCM (U-MV-FCM) [11] can search for the optimal number of clusters during the algorithm iteration process. These clustering algorithms combine the information in the image space to achieve a segmentation effect. However, they are sensitive to the number of initial centers and center points, and the segmentation effect of non-convex data is poor. Literature [12] constructs a robust K-nearest map to represent the structure of each image by graph theory. Then, maps in the same image domain are compared by map mapping, and changes are detected by the Markov co-segmentation model, thus improving the final detection performance. However, the processing speed is slow due to graph model construction and weight setting complexity, especially when processing large-scale images. The benefits and shortcomings of different image segmentation algorithm are shown in Table 1.

Traditional image segmentation methods have significant drawbacks, including sensitivity to noise and a lack of adaptive learning capabilities. In contrast, deep learning techniques, particularly neural networks, have become popular for image segmentation due to their effectiveness. For example, while backpropagation (BP) neural networks can handle nonlinear data, they often struggle with local minimum, impacting convergence with large datasets. Convolutional neural networks (CNNs) [13] enhance BP networks but require fixed input image sizes. Fully connected neural networks (FCNs) and recurrent neural networks (RNNs) provide better generalization but require extensive training datasets. The introduction of the channel space attention (CSA) and semantic guided attention (SGA) modules in dense channel space semantic guided attention UNet (DCSSGA-UNet) [14] selectively focuses on important features and reduces redundancy, thereby effectively bridging the semantic gap. Deep low-rank tensor embedding (DLTE) uses deep non-negative matrix factorization (NMF) to project high-dimensional data into the low-dimensional embedding space, thereby effectively capturing the complex nonlinear relationships in the data [15]. With the increasing scale of data, the literature proposes a brain tumor segmentation framework based on federated learning and U-Net, which improves the model performance while protecting data privacy through distributed multi-agency collaboration [16]. When fewer relevant training data are available, deep learning methods may experience high computational demands and inefficient training processes. In summary, traditional methods face issues like over-segmentation and noise sensitivity, while neural network-based approaches heavily rely on data and computing resources. Therefore, there is a need for methods that are computationally efficient yet accurate. Meta-heuristic algorithms emerge as viable alternatives for multi-threshold image segmentation due to their rapid convergence and high precision. For instance, particle swarm optimization (PSO) is effective but prone to local minimum. Enhancements like quantum operations can improve their performance [17]. In another study [18], bacterial foraging optimization (BFO) has shown robust results but may lack efficiency compared to other algorithms. Techniques like gray wolf optimization (GWO) and the cuckoo search have been applied to optimize multi-level thresholds, yielding better accuracy and speed [19]. In Ref. [20], the cuckoo algorithm (CS) is applied to image segmentation optimization based on recursive minimum cross-entropy, and experimental results show that its segmentation accuracy and speed are better than BFO, and its robustness is also improved. Ref. [21] uses the symbiotic biological algorithm (SBA) to process multi-threshold image segmentation. Ref. [22] made improvements by perfecting the three main steps of bacterial foraging optimization (BFO). Although the search speed is fast, it is easy to fall into the local optimal solution, and the global search ability could be more robust. BKAPI [23] addresses the premature convergence issue of the black-winged kite algorithm (BKA) in high-dimensional optimization problems by proposing an improved hybrid algorithm that integrates particle swarm optimization (PSO) and differential evolution (DE). This multi-strategy fusion effectively balances exploration and exploitation, improving convergence speed and computational accuracy. However, the hybrid mechanism of BKAPI is relatively complex, increasing the difficulty in implementation and parameter tuning. Ref. [24] cited in the literature uses PSO to optimize the dynamic model parameters of a pneumatic artificial muscle (PAM). This method demonstrates good performance in preliminary experiments, but its optimization process relies heavily on the global search capability of PSO, leading to limited convergence speed. Moreover, the experiments are based only on preliminary testing and do not involve complex or long-term operational scenarios, so the robustness and adaptability of the algorithm still require further verification. Ref. [25] integrates swarm intelligence algorithms with human-inspired electoral behaviors, proposing an improved PSO algorithm featuring a hierarchical structure and group interaction behaviors. By introducing competitive and supportive behavior mechanisms, the algorithm enhances the particle learning strategy and improves global search capability in the mid-optimization phase. In the later stages, a mutation mechanism is introduced to avoid premature convergence to local optima. However, although this mutation mechanism helps to maintain population diversity, it also introduces additional computational overhead, which may affect the algorithm’s real-time performance, and its impact on algorithm stability is not thoroughly analyzed. ROA [26] is a novel swarm intelligence optimization algorithm which is inspired by the foraging behavior of rats. However, the improvement in ROA mainly relies on the simulation of rat behavior, and the behavior model is still relatively empirical and simplistic, lacking theoretical support. Furthermore, although the algorithm shows improved convergence speed and accuracy, its adaptability to high-dimensional, dynamic, or constrained optimization problems is not clearly stated, so its generalization ability still requires further validation. MISWOA [27] proposes an improved whale optimization algorithm (WOA) named multi-swarm improved spiral whale optimization algorithm. MISWOA introduces an adaptive nonlinear convergence factor, variable gain compensation mechanism, adaptive weight strategy, and an improved spiral convergence strategy, thereby enhancing the algorithm’s performance in multiple aspects. However, although MISWOA shows good performance in various test scenarios, its sub-swarm division and cooperation strategies still depend on manual settings, lacking an adaptive adjustment mechanism for dynamic swarm structures. In addition to the swarm intelligence algorithms mentioned above, many emerging algorithms have gradually developed and been utilized in recent years.

**Table 1:** The benefits and shortcomings of different image segmentation algorithm.

Algorithms	Benefits	Shortcomings
OTSU [3]	performs well in simple scenes	performs worse in complex backgrounds
RAV-WOA [4]	optimized the original OTSU method	low accuracy
FOA-OTSU [5]	the search speed is fast	many noise points
K-Means [6,7]	principle is relatively simple, easy to implement and has a fast convergence speed	be relatively sensitive to noise and outliers
FCM [8,9]	improves the inclusion and accuracy of segmentation	sensitive to the number of initial centers and center points
W-MV-KM-L2 [10]	emphasize the importance of weighted multi-view learning	application scenarios are limited
U-MV-FCM [11]	the number of clusters does not need to be given prior	performance when dealing with data with significant differences in view quality needs to be further explored
MBF-OTSU [12]	easy to implement	processing speed is slow due to graph model construction
DCSSGA-UNet [14]	can effectively detect the variability of objects	robustness in processing images containing noise and artifacts was not mentioned
DLTE [15]	learn more reasonable local geometric structures of data in the deeply embedded space	complex and has a long running time
Ref. [16]	problems of data privacy and security in the task of brain tumor segmentation have been solved	generalization ability and communication overhead of the algorithm were not mentioned
R-MCE-CS [20]	robustness is improved	optimization result is not good enough
Ref. [21]	the search speed is fast	easy to fall into the local optimal solution, and the global search ability could be more robust

**Figure 1:**
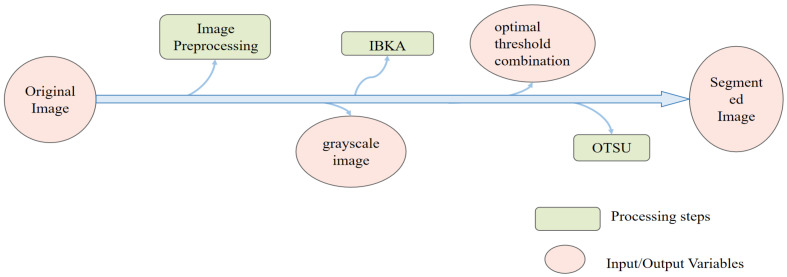
IBKA-OTSU image segmentation framework.

This paper proposes an improved black-winged kite algorithm (IBKA) for multi-threshold segmentation, which is a bio-inspired metaheuristic optimization method that emulates the foraging and attacking behaviors of BKA, a species of bird of prey. The proposed algorithm incorporates several key enhancements in the following aspects: population initialization, aggressive behavior modeling, migration behavior, and reverse learning strategy. Specifically, SPM chaotic mapping is introduced to improve the diversity and global search capability of the initial population; the attack behavior formula is modified to an exponential decay form to enhance the convergence speed and precision; migration behavior is inspired by the sparrow search algorithm (SSA) to further enrich the exploration diversity; and reverse learning strategy and adaptive T-distribution strategy are integrated to effectively balance exploration and exploitation during the optimization process. These modifications are all grounded in the observation and mathematical abstraction of natural biological behaviors, which is a fundamental principle in the field of biomimetics—that is, to draw inspiration from nature to design more efficient, robust, and adaptive computational models. The overall goal of the IBKA-OTSU image segmentation framework is to optimize the threshold selection by maximizing inter-class variance, thereby achieving improved performance in terms of accuracy, precision, and computational efficiency. The IBKA-OTSU image segmentation framework proposed in this paper is shown in Figure 1.

Experiments indicate that the proposed algorithm effectively addresses the issues of extensive computation and long processing times associated with traditional segmentation methods. The results from random remote sensing image segmentation demonstrate that the method introduced in this paper is more efficient than previous approaches. It is less likely to encounter the problem of “insufficient segmentation”, and it shows significant improvements in anti-noise performance and accuracy. The contributions of this paper are as follows:

**Improved Algorithm (IBKA-OTSU)**: The improvements are defined through a thorough analysis of the original BKA algorithm, enhancing the four key steps of the black-winged kite’s approach. The IBKA-OTSU is proposed to maximize inter-class variance as per the OTSU method, and a flowchart illustrating the IBKA-OTSU algorithm is provided.
**Validation of Improved Algorithm**: This paper presents experimental results using the CEC2019 test function to validate the effectiveness of the improved algorithm. The research compares the performance of the IBKA algorithm with classical heuristic algorithms and more recent methodologies. The quantitative analysis results demonstrate that IBKA offers superior anti-noise performance and accuracy compared to other methods.
**Practicality and Universality of the Algorithm**: The IBKA-OTSU algorithm’s practicality and universality are verified through its application to remote sensing images. Six randomly selected images from the ISPRS Potsdam dataset are analyzed. When compared to classical remote sensing image segmentation methods, the results confirm the superiority and practicality of the proposed algorithm. Finally, the thesis is summarized, and prospects are discussed.

## 2. Related Works

### 2.1. OTSU

**Figure 2:**
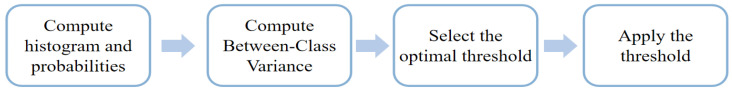
OTSU algorithm flowchart.

The threshold segmentation method determines a particular threshold value of the image according to the size of the gray value of each pixel in the image and the threshold value of the comparison to segmentation. The threshold value is set manually according to the valley of the grayscale histogram. Threshold segmentation by the number of thresholds selected is divided into single-threshold segmentation and multi-threshold segmentation. Multi-threshold segmentation is a more classic image segmentation method, but the lack of universality is due to the complexity of manually setting the threshold. Therefore, the automatic threshold segmentation method OTSU proposed by Nobuyuki Otsu is the more commonly used image segmentation method, also known as maximizing inter-class variance segmentation. The flowchart of the OTSU algorithm is shown in Figure 2:

The OTSU multi-threshold segmentation method is suitable for any number of threshold segmentation, let the image size be M∗N and the image gray level range be [0 , L−1], $n_{i}$ is the number of pixels of image gray level i. Then the probability of occurrence of gray level i is $P_{i}=\frac{n_{i}}{\left(M*N\right)}$, a set of thresholds $\left[t_{1},t_{2},\ldots ,t_{n-1}\right]$ classifies the image into n classes, and the probability of occurrence of each class is $\left[P_{0},P_{1},\ldots {,P}_{n-1}\right]$. The corresponding mean is $\left[\mu_{0},\mu_{1},\ldots ,\mu_{n-1}\right]$, and the variance is $\left[\delta_{0}^{2},\delta_{1}^{2},\ldots ,\delta_{n-1}^{2}\right]$, then for each class k∈[0 , L−1] there are:

$$P_{k}=\sum_{i=t_{k}}^{t_{k+1}-1}P_{i}$$

(1) $$u_{k}=\frac{1}{P_{k}}P_{k}\;\sum_{i=t_{k}}^{t_{k+1}-1}{iP}_{i}$$

$$\delta_{k\;}^{2}=\sum_{i=t_{k}}^{t_{k+1}-1}{\left(i-u_{k}\right)}^{2}\frac{P_{i}}{P_{k}}$$

where the intra-class variance of this grayscale image can be expressed as:

(2) $$\sigma_{b}^{2}=\sum_{i=0}^{n-1}P_{k}{\left(\mu_{k}-\mu \right)}^{2}$$

The optimal threshold is when Equation (2) gets the maximum value. In this paper, the idea of the OTSU algorithm is introduced, and the intra-class variance is used as the fitness function of the improved swarm intelligent optimization algorithm to obtain the optimal threshold value and further use the multi-threshold segmentation method to process the image.

### 2.2. The Black-Winged Kite Algorithm

The black-winged kite algorithm (BKA) [28] was proposed by J Wang et al. in 2024, which simulates the behaviors, such as aggression and migration, of the black-winged kite, a species. According to the different survival strategies of the population, the algorithm is specifically divided into three phases: population initialization, attacking behavior, and migratory behavior.

#### 2.2.1. Initialization Phase

BKA first randomly generates an initialized population based on the search space, setting pop to be the number of potential solutions and dim to be the dimension size of the given problem, then for the $i^{th}$ proposed solution $X_{i}$ has:

(3) $$X_{i}={BK}_{lb}+rand\times \left({BK}_{ub}-{BK}_{lb}\right),\;\;i\in [1,pop]$$

where i is an integer between $\left[1,\;pop\right]$, ${BK}_{ub}$ and ${BK}_{lb}$ denote the upper and lower bounds of the $j^{th}$ dimensional black-winged kite, respectively, and rand is a random number between $\left[0,\;1\right]$. Then the position of each black-winged kite in the overall initial population can be represented by the matrix:

(4) $$BK=\left[\begin{array}{ccccc} {BK}_{1,1} & \ldots & {BK}_{1,j} & \ldots & {BK}_{1,dim}\\ \vdots & \ldots & \vdots & \ldots & \vdots \\ {BK}_{i,1} & \ldots & {BK}_{i,j} & \ldots & {BK}_{i,dim}\\ \vdots & \ldots & \vdots & \ldots & \vdots \\ {BK}_{pop,1} & \ldots & {BK}_{pop,j} & \ldots & {BK}_{pop,dim} \end{array}\right]$$

where BK is the population of black-winged kites and ${BK}_{i,j}$ is the value of the $j^{th}$ dimensional variable specified by the $i^{th}$ proposed solution.

#### 2.2.2. Attacking Behavior

After detecting the prey, the black-winged kite in flight first adjusts its wings and tail according to the wind speed, hovers in the air to observe the prey, and then quickly dives to attack. Therefore, in mathematical modeling, the strategy is divided into two different attack behaviors, and the mathematical expression for the behaviors is as follows.

(5) $$y_{t+1}^{i,j}=\left\{\begin{array}{c} y_{t}^{i,j}+n\left(1+\sin\left(r\right)\right)\times y_{t}^{i,j},p<r\\ y_{t}^{i,j}+n\times \left(2r-1\right)\times y_{t}^{i,j},else \end{array}\right.$$

(6) $$n=0.05\times e^{-2\times {\left(\frac{t}{T}\right)}^{2}}$$

where $y_{t}^{i,j}$ and $y_{t+1}^{i,j}$ are the positions of the $j^{th}$ variable of the $i^{th}$ black-winged kite during the $t^{th}$ iteration and ${t+1}^{th}$ iteration, r is a random number between $\left[0,\;1\right]$, p is a constant with the value of 0.9. T is the total number of iterations, and t is the current number of iterations [28].

#### 2.2.3. Migration Behavior

As birds, black-winged kites migrate due to climate change and food availability. They usually migrate in groups where a leader kite guides other individuals’ movement direction. During migration, black-winged kites share information through visual and acoustic means, and the algorithm can be used to improve the overall search efficiency of the population through the exchange of information between individuals, such as the sharing of location and fitness values. black-winged kites adjust to changes in the environment during migration, such as avoiding obstacles or searching for food. Therefore, an adaptive adjustment mechanism is introduced into the algorithm, which enables individuals to dynamically adjust their search strategy to adapt to the complex search space. The mathematical expression for the migration stage is:

(7) $$y_{t+1}^{i,j}=\left\{\begin{array}{c} {\;\;\;y}_{t}^{i,j}+C(0,1)\times (y_{t}^{i,j}-L_{t}^{j}),\;\;\;\;\;\;\;\;\;\;\;\;\;\;\;\;F_{i}<F_{ri}\\ y_{t}^{i,j}+C(0,1)\times (L_{t}^{j}-m\times y_{t}^{i,j}),\;\;\;\;\;\;\;\;\;\;\;\;\;\;\;\;\;\;\;else \end{array}\right.$$

(8) $$m=2\times sin\left(r+\frac{\pi }{2}\right)$$

where $L_{t}^{j}$ denotes the leading kite of the black-winged kite in the $j^{th}$ dimension variable in the $t^{th}$ iteration so far. $y_{t}^{i,j}$ and $y_{t+1}^{i,j}$ are the positions of the $i^{th}$ black-winged kite in the $j^{th}$ variable during the $t^{th}$ iteration and ${t+1}^{th}$ iteration. r is a random number between $\left[0,\;1\right]$, and $F_{i}$ denotes the $j^{th}$ dimension of the $i^{th}$ black-winged kite in the $t^{th}$ iteration of its current position. $F_{ri}$ denotes the fitness value of the random position in the $j^{th}$ dimension obtained by the $i^{th}$ black-winged kite in the $t^{th}$ iteration. C(0,1) stands for the Cauchy mutation, and the one-dimensional Cauchy distribution is a continuous probability distribution with two parameters. The following equation is the mathematical expression for the probability density function of the one-dimensional Cauchy distribution:

(9) $$f\left(x,\delta ,\mu \right)=\frac{1}{\pi }\frac{\delta }{\delta^{2}+{\left(x-\mu \right)}^{2}\;},-\infty <x<\infty$$

When δ=1,μ=0 in Equation (9), its probability density function will be in standard form:

(10) $$f\left(x,\delta ,\mu \right)=\frac{1}{\pi }\frac{1}{x^{2}+1},-\infty <x<\infty$$

Suppose the fitness value of the current population is less than the fitness value of the random population. In that case, it reflects that the leader is leading in the wrong direction, and the leader will give up leadership and join the migratory population. On the contrary, if the fitness value of the current population is greater than the fitness value of the random population, it will guide the population until it reaches its destination. This strategy allows for a dynamic selection of good leaders to ensure migration success. Compared with the emerging group intelligence algorithms in recent years, BKA is characterized by fast convergence and high efficiency. However, it also faces problems such as low accuracy of group intelligence algorithms and easy falling into local optimization. In this paper, we improve the traditional BKA to address the above problems and further improve the convergence accuracy and optimization search speed of BKA.

## 3. An Adaptive Multi-Threshold Image Segmentation Method Based on Improved Black-Winged Kite Algorithm (IBKA-OTSU)

**Figure 3:**
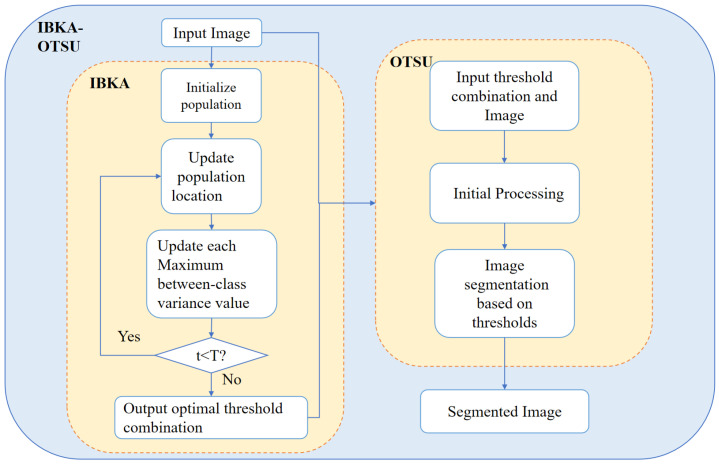
Flowchart of IBKA-OTSU algorithm.

Given that the BKA has the problems of quickly falling into a local optimal solution and slow convergence, this paper optimizes the multi-threshold segmentation algorithm by improving BKA. Firstly, the SPM chaotic mapping was added to initialize the population; change the original update formula to exponential decay during the BKA attack behavior stage; integrate the sparrow search strategy in the migratory behavior stage; in addition, the reverse learning strategy has been added. The above improvements are made to enhance the accuracy rate, optimization precision and segmentation effect of the algorithm. It proposes the IBKA-OTSU algorithm by improving the four critical steps of BKA, including population initialization, attacking behavior, migration behavior, and inverse learning strategy. Then, the maximum inter-class variance is used as the fitness function to improve the BKA to get the optimal threshold combination of the multi-threshold segmentation algorithm to segment the remote sensing images to improve the algorithm’s accuracy, optimization searching precision, and segmentation effect. The flow chart of the IBKA-OTSU algorithm proposed in this paper is shown in Figure 3.

### 3.1. SPM Chaotic Mapping

**Figure 4:**
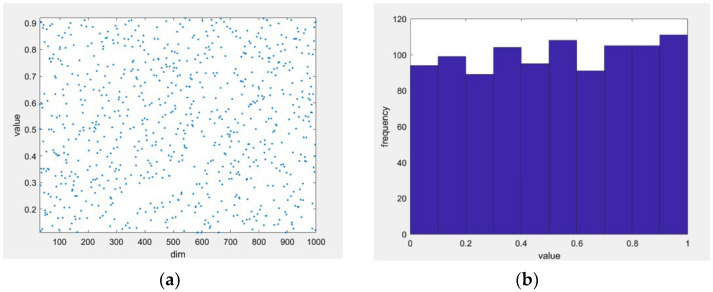
Piecewise mapping. (**a**) the chaotic sequence generated by SPM. (**b**) the frequency of occurrence of each value.

The selection of the initial population location can directly affect the search efficiency, global search ability, and the ability to avoid falling into the local optimal solution. An excellent initial population distribution can help the algorithm cover the entire search space and increase the chance of finding the global optimal solution. On the contrary, if the initial population location is not selected correctly, the population may be concentrated in a particular area of the search space, thus falling into the local optimal solution and affecting the algorithm’s performance. This paper uses SPM chaotic mapping to generate the initial population location to make the initial population distribution more uniform. The mathematical expression of SPM chaotic mapping is as follows:

(11) $$x\left(t+1\right)=\left\{\begin{array}{c} mod(\frac{x\left(t\right)}{\eta }+\mu \sin\left(\pi x\left(t\right)+r,1\right),\;\;0\leq x\left(t\right)<\eta \\ mod(\frac{\frac{x\left(t\right)}{\eta }}{0.5-\eta }+\mu \sin\left(\pi x\left(t\right)+r,1\right),\;\;\eta \leq x\left(t\right)<0.5\\ mod(\frac{\frac{1-x\left(t\right)}{\eta }}{0.5-\eta }+\mu \sin\left(\pi \left(1-x\left(t\right)\right)+r,1\right),\;\;0.5\leq x\left(t\right)<1-\eta \\ mod(\frac{\left(1-x\left(t\right)\right)}{\eta }+\mu \sin\left(\pi \left(1-x\left(t\right)\right)+r,1\right),\;\;1-\eta \leq x\left(t\right)<1 \end{array}\right.$$

where r is the random number between $\left[0,\;1\right]$, η and μ take the values of (0,1). The sequence generated by SPM chaotic mapping is a nonlinear dynamic system with ergodic and stochastic properties, which can enhance diversity, avoid periodicity and determinism, improve global search ability, and improve the stability and convergence of the algorithm. Compared with the pure random generation method, chaotic mapping can avoid periodicity and determinism and produce richer search space coverage. In this paper, when η=0.5, μ=0.5, the sequence generated by SPM chaotic mapping is shown in Figure 4 below.

The left figure (a) shows the chaotic sequence generated by SPM, and the right figure (b) shows the frequency of occurrence of each value, which shows that the SPM chaotic mapping is capable of generating uniform chaotic points within the given range, which satisfies the required requirements for initializing the population.

### 3.2. Improved Attack Behavior Formula into Exponential Decay Form

The formula of the BKA attack phase determines the search performance of the algorithm to a large extent because it is the basis of the algorithm’s global or local search. The formula of the traditional BKA attacking behavior above is only a simple processing of the previous position and cannot be dynamically adjusted according to the number of iterations. As a result, the step size in the early stage is too small to explore the entire search space entirely, and the optimal solution is easily skipped in the later behavior. Therefore, based on the original Formula (5), the formula is transformed into exponential decay. The improved mathematical representation is as follows:

(12) $$y_{t+1}^{i,j}=\left\{\begin{array}{c} y_{t}^{i,j}\times exp\left(\frac{-t}{r\times T}\right)\times y_{t}^{i,j},\;\;p<r\\ y_{t}^{i,j}+n\times \left(2r-1\right)\times y_{t}^{i,j},\;\;else \end{array}\right.$$

where t is the current number of iterations, T is the total number of iterations, and r is the random number between $\left[0,\;1\right]$, p is a random number between $\left[0,\;1\right]$. The comparison of p with r and the selection strategy demonstrate the adaptive ability of the algorithm. The advantage of replacing the traditional formula with the exponential decay form mentioned above is that it can dynamically adjust the exploration and development balance of the algorithm. By gradually reducing the search step size and weight, it helps the algorithm to conduct an extensive search in the initial stage to explore the possible region of the global optimal solution more effectively. This process gradually narrows down to a more accurate local search in the later stage, improving both the solution’s accuracy and the algorithm’s convergence speed, ultimately enhancing the stability and optimization effect of the BKA algorithm.

### 3.3. Migration Behavior of the BKA with a Sparrow Search Algorithm

In this paper, the sparrow search algorithm is used to improve the migration behavior strategy of BKA and solve the problem of the low accuracy of the BKA. The sparrow search algorithm [29] was developed by Donghua University Xue et al. in 2020. A new swarm intelligent optimization algorithm is proposed. The algorithm seeks a solution to the optimization problem by simulating the foraging and anti-preying behavior of sparrows. In this paper, the sparrow position formula in SSA, where the periphery is aware of the danger, is replaced by the migration behavior formula in BKA. The improved formula is as follows:

(13) $$y_{t+1}^{i,j}=\left\{\begin{array}{c} {\;\;\;y}_{t}^{i,j}+C(0,1)\times (y_{t}^{i,j}-L_{t}^{j}),\;\;\;\;\;\;F_{i}<F_{ri}\\ L_{t}^{j}+r_{1}\times |{ybest}_{t}^{i,j}-L_{t}^{j}|,\;\;\;\;\;\;\;\;\;\;\;else \end{array}\right.$$

where $r_{1}$ is the random number between $\left[0,\;1\right]$, and ${ybest}_{t}^{i,j}$ is the historical optimal value of the position of the $j^{th}$ variable of the $i^{th}$ black-winged kite in the $t^{th}$ iteration. Since the second subformula in the formula makes it easy to ignore the historical optimal solution of the current scheme and replace it with the above formula, the position is updated according to the current optimal value and the individual historical optimal value, which can improve the possibility of finding a better solution.

### 3.4. Reverse Learning Strategy and Adaptive T-Distribution Strategy

This paper integrates the reverse learning strategy into BKA to expand the scope of the solution to enable individuals to better find the optimal solution. The mathematical expression of reverse learning is as follows:

$$\;b={\;(T-\frac{t}{T})}^{t}$$

(14) $${LB}_{t}^{j}={BK}_{ub}+r_{2}⊕\left({BK}_{lb}-L_{t}^{j}\right)$$

$${\;y}_{t+1}^{i,j}={LB}_{t}^{j}+b⊕\left(y_{t}^{i,j}-{LB}_{t}^{j}\right)$$

where b represents the information exchange control parameter, ${LB}_{t}^{j}$ is the reverse solution of the optimal solution in the $t^{th}$ iteration. By introducing the reverse symmetric point of the optimal solution, the diversity of the search space is increased, and the global search ability of the algorithm is improved.

This paper uses the T-distribution mutation operator with the number of iterations as the degree of freedom parameter to perturb the individual position to further improve the optimization performance of the algorithm. The mathematical expression of the adaptive T-distribution strategy is shown as follows:

(15) $$y_{t+1}^{i,j}=y_{t}^{i,j}+y_{t}^{i,j}\times t(t)$$

The random disturbance term $y_{t}^{i,j}\times t(t)$ is added to the formula, which improves randomness while making full use of the current position information so that the algorithm has a better global development ability in the early iteration stage. With the increase in iteration t, the T-distribution gradually moves closer to the Gaussian distribution, which is conducive to enhancing the algorithm’s local search ability and convergence speed. It is obviously inappropriate to use the above two strategies for each individual without discrimination, but it will increase the algorithm’s running time. Therefore, this paper adopts the selection probability p to adjust the application of the reverse learning and adaptive T-distribution strategies. The specific selection scheme is as follows: p is a random number between $\left[0,1\right]$. If p<0.5, the reverse learning strategy is selected; otherwise, another strategy is chosen. This improved algorithm provides a fast way to locate high-quality solutions in the search space, which helps to accelerate the algorithm’s convergence.

The pseudo-code of the improved IBKA algorithm based on the above steps is shown below (Algorithm 1).
**Algorithm 1. Improved black-winged kite algorithm.****Input:** The population size *pop*, variable dimension *dim*, and maximum number of iterations *T*.
**Output:** The best quasi-optimal solution Fbest obtained by IBKA for the given optimization problem.1. Initialization phase
2. Initialization of the position of black-winged kites using (11) and evaluation of the objective function.
3. Calculate the fitness value of each Black-winged kite.
4. **For**
*t* = *1*: *T*
5.   **For**
i = *1*: *pop*
6.      *Attacking behavior*
7.      Update $i^{th}$ population member use (12).
8.      *Migration behavior* 
9.      Calculate the probability density function of the Cauchy distribution using (10).
10.    Update $i^{th}$ population member using (13).
11.    *Backward Learning Strategy and adaptive T-distribution strategy*
12.    **if**
p<0.5

13.        Using reverse learning strategy according to Formula (14).
14.    **else**
15.        Using reverse learning strategy according to Formula (15).
16.     end if
17.     *Select the best individual*
18.     $if\;y_{t+1}^{i,j}<L_{t}^{j}$
19.   $Xbest=y_{t+1}^{i,j},\;Fbest={f(y}_{t+1}^{i,j})$
20.   else
21.        $Xbest=L_{t}^{j}$, $Fbest=f(L_{t}^{j})$
22.     **end if**
23.   **end for**
*i* = *1*: *pop*
24. **end for**
*t* = *1*: *T*
25. Return *Xbest* and *Fbest*

### 3.5. Adaptive Multi-Threshold Segmentation Method Based on IBKA

Multi-threshold segmentation methods are simple to operate but have poor optimization-seeking ability, resulting in poor subsequent image segmentation. To get the optimal combination of thresholds to achieve good segmentation, this paper adopts the idea of using the maximum interclass variance of the image in OTSU in Section 2.1. And use Formula (2) as the fitness function of the optimization algorithm and the IBKA to perform the threshold optimization to get the optimal threshold value.

The multi-threshold segmentation method combined with IBKA can avoid the drawbacks of traditional threshold segmentation, which requires setting thresholds or finding thresholds based on histogram analysis manually. It can quickly and effectively find the optimal combination of solutions and solve the problem of poor segmentation accuracy and precision that exists when multi-threshold segmentation is applied alone. The pseudo-code of the proposed IBKA-OTSU algorithm is shown below (Algorithm 2).
**Algorithm 2. IBKA-OTSU algorithm.****Input:** The original image.
**Output:** Segmentation result image.1. Image preprocessing
2. Define the value of the population size *pop*, variable dimension *dim*, and maximum number of iterations *T*.
3. Start the IBKA iterative loop.
4.      Algorithm 1.
5. Obtain the *Xbest* and *Fbest*.
6. *Xbest* is the optimal combination of parameters.
7. Start OTSU. 
8. Segmentation result image.

## 4. Comparative Experimental Analysis of IBKA-OTSU Algorithms

### 4.1. Test Environment and Functions

This part aims to evaluate the effectiveness and practicability of the improved algorithm by comparing it with other swarm intelligence algorithms. The experimental environment is MATLAB 2022b, and the test platform processor is i7-13620H (2.4 GHz). To test the optimization accuracy and running time of the IBKA. This paper uses the CEC2019 test function [30] to conduct comparative experiments. The CEC2019 test set has 10 single-objective test functions. The variable dimension and search interval of each function are different, so the solving ability and stability of different algorithms on the same optimization problem can be compared, and the effect of optimization algorithms in solving practical problems can be evaluated [30]. This test set contains 10 single-objective test functions, which can test the convergence performance of the algorithm and the balance ability between global exploration and local development of the decision space. Therefore, it can effectively evaluate the performance of the algorithm and is one of the most widely used and classic test sets. Moreover, the test function in CEC2019 is consistent with the single objective function in remote sensing image segmentation and is applicable to the remote sensing image segmentation task. All the test functions in CEC2019 solve the minimization problem; the optimal value is 1, and D is the dimension, as shown in Table 2.

### 4.2. Comparative Performance Analysis

**Figure 5:**
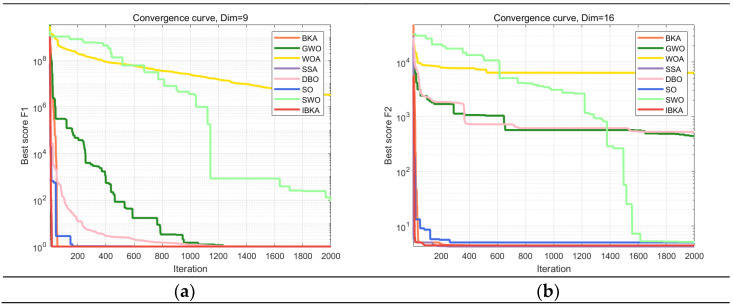

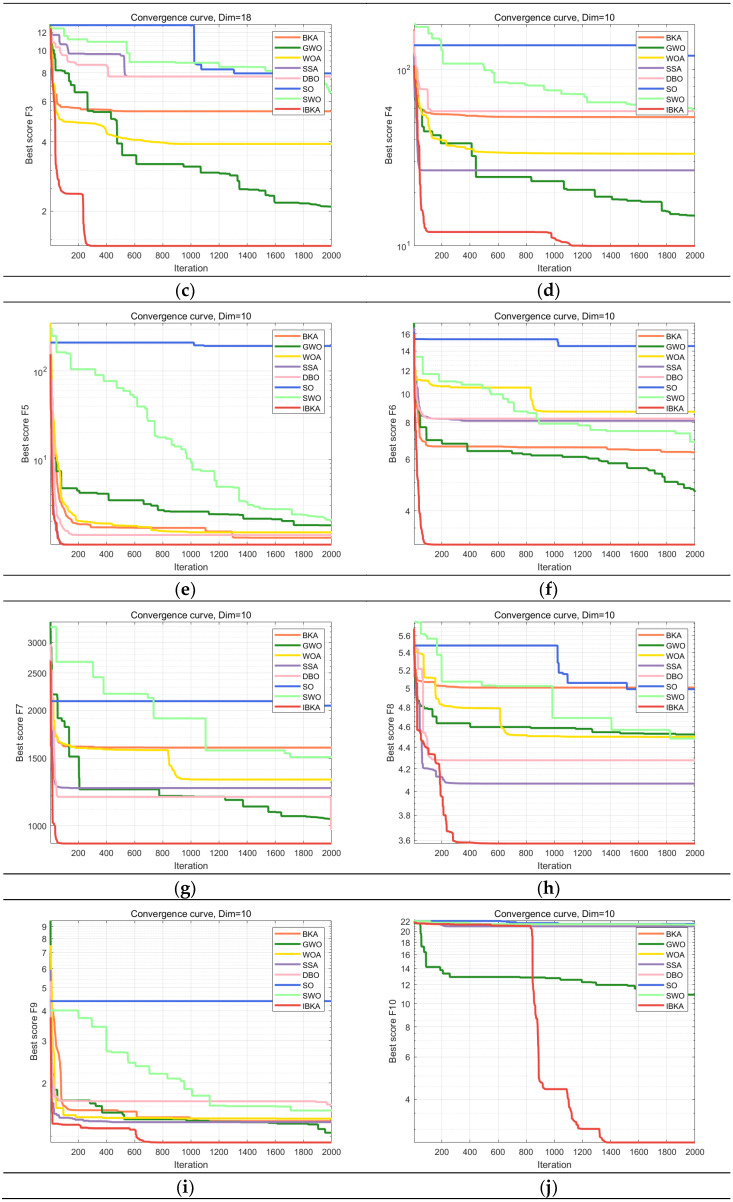
(**a**) The convergence curves of F1, (**b**) the convergence curves of F2, (**c**) the convergence curves of F3, (**d**) the convergence curves of F4, (**e**) the convergence curves of F5, (**f**) the convergence curves of F6, (**g**) the convergence curves of F7, (**h**) the convergence curves of F8, (**i**) the convergence curves of F9, (**j**) the convergence curves of F10.

The improved IBKA in this paper is compared with the classical heuristic algorithms in recent years: grey wolf optimizer (GWO) [31], whale optimization algorithm (WOA) [32], sparrow search algorithm (SSA) [29], dung beetle optimizer (DBO) [33], and new algorithms proposed in recent years: snake optimization (SO) [34] and spider wasp optimizer (SWO) [35]. Moreover, it also compared with the original BKA before the improvement. Each algorithm’s population pop is set to 30, and the maximum number of iterations T is set to 2000. The optimal value evaluates the algorithm’s performance, as well as the worst value and the average value. To reduce errors and ensure the accuracy of the experiment, each function is independently run 50 times. The performance of different optimization algorithms on CEC2019 is shown in Table 3. The bold reflects the optimal solution in the comparison results. To directly observe the convergence speed of each algorithm, the convergence iteration curve corresponding to 10 test functions is shown in Figure 5.

From the test results of F1, F3, and F10, the IBKA can find the optimal values of these three functions: 1. In the other seven test functions, IBKA can also find more minor results than other heuristic optimization algorithms. Although the optimal value can be found for all algorithms except WOA for the F1 function, the average value of IBKA is smaller than other algorithms, and the average value of all functions is better than other algorithms. It shows that the improved IBKA has a stable effect and is of higher quality. Observing the iteration curve shows that when t = 1800 to 2000 in the late iteration, there is still a heuristic algorithm convergence. However, IBKA can quickly converge to the optimal value within 200 times in most functions. Although IBKA still converges for F8 and F10 in the later iteration period, since other algorithms cannot reach the optimal value and IBKA can find the minimum value of F10, the result of F8 is much smaller than that of other functions, indicating that this algorithm can jump out of the local optimal solution. For F1 and F2, IBKA converges to the optimal value in less than 50 iterations. It is sufficient to show that IBKA has a fast convergence rate. The experiments of IBKA and other algorithms with ten functions in the CEC2019 test set show that IBKA has the characteristics of good robustness, high precision, and fast convergence speed and is an algorithm with comprehensive solid ability.

## 5. Application Experiment of IBKA-OTSU on Remote Sensing Images

The validity and stability of the proposed algorithm are explained in the previous part. To further prove the practicability of the proposed algorithm, images from the ISPRS Potsdam remote sensing dataset are selected for experiments. With the development of remote sensing technology, the accuracy and spatial resolution of remote sensing images are constantly improved, bringing multi-spectral images. RGB remote sensing images are applied to large-scale images, and rich color space further increases the difficulty of image segmentation [36]. To adapt to the rapid development of remote sensing technology, segmentation technology has ushered in higher challenges and requirements [37]. Compared with conventional images, remote sensing images have the characteristics of different acquisition angles, small image targets, complex image backgrounds, foreground-background imbalance, etc., which inevitably bring the problems of extensive calculation, long time, and low segmentation accuracy to remote sensing image processing. This makes it difficult to accurately locate and recognize foreground features of remote sensing images, resulting in missegmentation of small and medium targets and edge information.

Typical remote sensing image segmentation techniques include the threshold method, edge detection method, region extraction method, and watershed algorithm based on morphology. These methods can achieve the purpose of segmentation, but the last three have limitations, such as low precision, high noise, and edge sensitivity. The threshold segmentation method is practical, easy to implement, and not prone to “insufficient segmentation”. According to the selection of different thresholds, there are a variety of threshold segmentation methods based on different entropy thresholds, including minimum cross entropy [38,39], Renyi entropy threshold [40,41], Shannon entropy [42,43], fuzzy entropy [44,45] and multi-threshold segmentation method (OTSU) based on maximum inter-class variance [46,47,48]. In this paper, the threshold number is 5 for the experiment.

### 5.1. Remote Sensing Image Database

**Figure 6:**
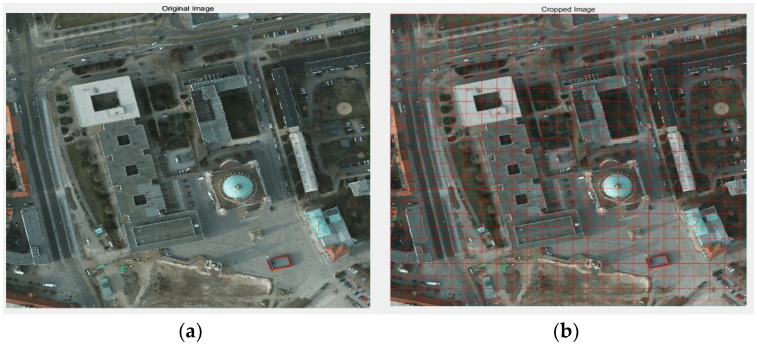
(**a**) Original image; (**b**) cropped image.

The ISPRS Potsdam database, a dataset commonly used in remote sensing imagery, shows that Potsdam has large building blocks, narrow streets, and dense settlement structures. There are 38 Potsdam images in the dataset, and the pixel value of each image is 6000×6000. Because the image is too large, some effective information will be lost during segmentation. In this paper, experiments are conducted by randomly selecting the trimmed data to demonstrate the generalization ability and universality of the proposed algorithm. This paper cuts the original image into 256 × 256 pictures, and the cutting effect is shown in Figure 6.

### 5.2. Image Preprocessing

**Figure 7:**
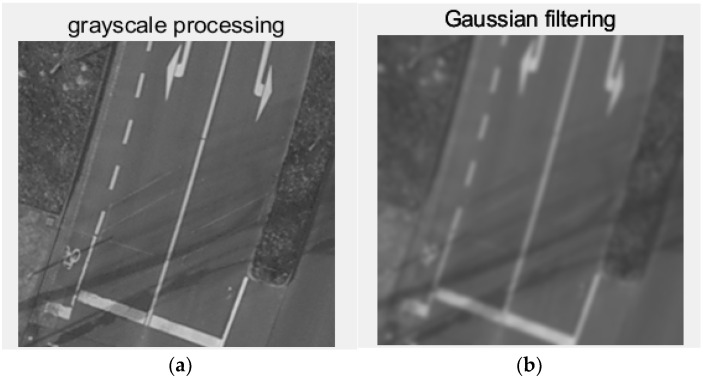
(**a**) Grayscale image; (**b**) Gaussian filtered image.

Before performing image segmentation, it is important to preprocess the original image through techniques such as gray-scaling and filtering. This preprocessing helps unify image features, smooth the image, reduce the influence of noise, and enhance the effectiveness of the subsequent segmentation. In this case, the original remote sensing image is processed using gray-scaling and Gaussian filtering. These methods remove color information and emphasize the structure and texture of the image, allowing useful information to be retained while unnecessary details and noise are minimized. As a result, the image segmentation process becomes more accurate and efficient. The image after gray-scaling and Gaussian filtering is shown in Figure 7 below:

### 5.3. Application of IBKA-OTSU in Remote Sensing Datasets

**Figure 8:**
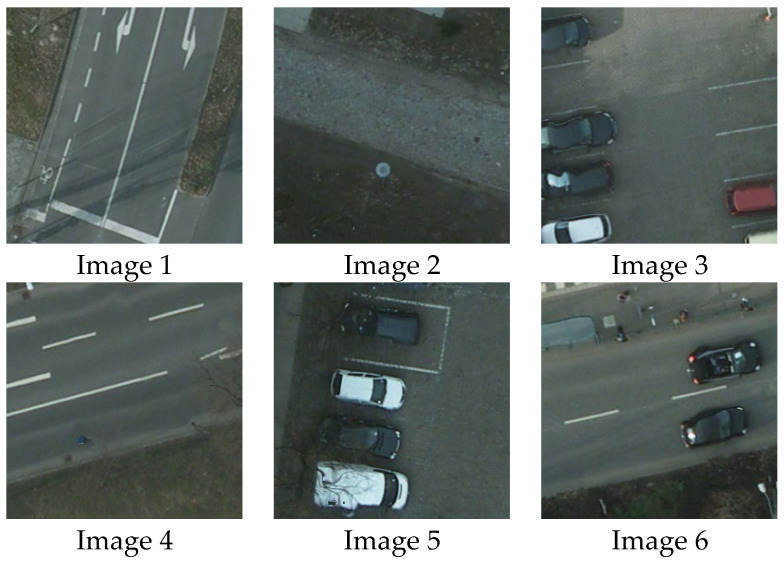
Original images.

In this experiment, six remote sensing images were randomly selected from the ISPRS Potsdam dataset, and the effectiveness and superiority of this method were proved. The original Image is shown in Figure 8. Images 1–6 are the 256 × 256 images after cutting, and the images after cutting are selected from RGB2_10_68, RGB3_10_57, RGB4_14_6, RGB5_12_29, RGB6_11_115, and RGB7_13_76, respectively.

**Figure 9:**
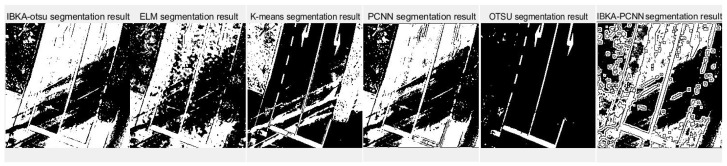
Segmentation results of image 1 by different algorithms.

**Figure 10:**
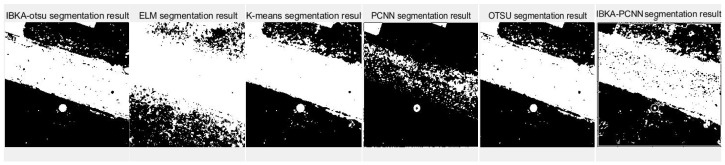
Segmentation results of image 2 by different algorithms.

**Figure 11:**
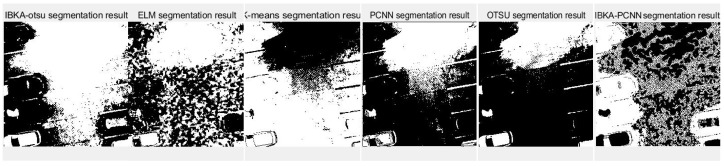
Segmentation results of image 3 by different algorithms.

**Figure 12:**
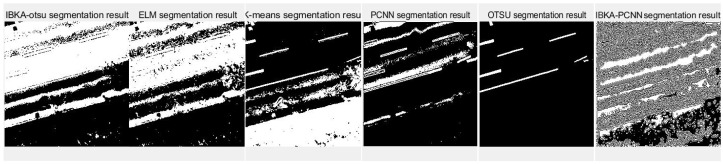
Segmentation results of image 4 by different algorithms.

**Figure 13:**
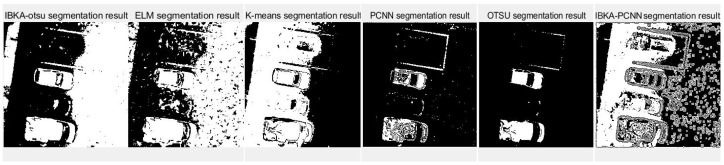
Segmentation results of image 5 by different algorithms.

**Figure 14:**
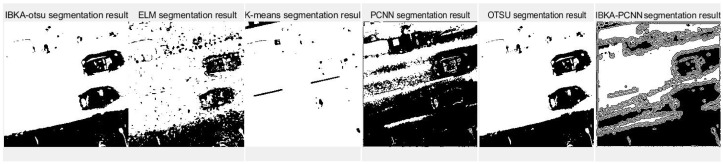
Segmentation results of image 6 by different algorithms.

The remote sensing image segmentation method proposed in this paper is compared with several commonly used and traditional image segmentation methods such as OTSU, PCNN, K-means clustering, and extreme learning machine (ELM), as well as improved traditional image segmentation methods proposed in recent years. Fan et al. [36] proposed in 2022 an improved sine and cosine algorithm to optimize remote sensing image segmentation parameters of PCNN. In this experiment, IBKA was used to optimize PCNN for remote sensing image segmentation, and the segmentation results are shown in Figure 9, Figure 10, Figure 11, Figure 12, Figure 13 and Figure 14 below.

Firstly, it can be seen from the above six segmentation results that the remote sensing image segmentation results optimized by the pulse-coupled neural network using IBKA contain many noise points. Although the optimization measures for the meta-heuristic algorithm and PCNN are proposed in the literature [36], there is still much noise in the segmentation results of the above remote sensing images, as well as the phenomenon of “under-segmentation” or “over-segmentation”. For complex images such as medical and remote sensing images with complex backgrounds, significant differences, uneven image feature density, etc. The segmentation method based on PCNN is not applicable. The IBKA-OTSU has a good effect, which can segment the road and background well, and the details are precise from the segmentation result of image 1 in Figure 9. Both the ELM and the IBKA-PCNN have many noise points; the ELM has under segmentation, some grassland is not correctly identified as the foreground, and the IBKA-PCNN also has under segmentation. On the other hand, K-means and OTSU have the problem of over-segmentation, ignoring the primary information, and identifying redundant road sign information. The OTSU is not fine enough in detail processing, and its edge is rough. The information in image 2 is clear; the road area segmented by the IBKA-OTSU is prominent, and the foreground and background separation effect is also significant. In the ELM method, part of the road area is not correctly identified as the foreground, there is an under-segmentation phenomenon, and more details are lost. K-means has more noise points and missegmentation areas, especially in the transition between the road edge and the background, which needs to be more accurate. The over-segmentation of the PCNN and the IBKA-PCNN is evident; more noise points are in the background, and the segmentation results are more complicated.

Although the original images of image 3 and image 5 are similar due to the different light sources, image 3 has some light information that will affect the segmentation results. As seen in Figure 11, the segmentation edge of IBKA is complete, and it can accurately segment vehicle information on the exit, and there are fewer internal noise points. The segmentation effect of other methods could be better, especially PCNN, K-means, and OTSU methods, which appear to be the phenomenon of over-segmentation, and the shadow area on the road surface is also segmented. Image 4 and image 6 also show that IBKA-OTSU has the best segmentation effect. In contrast, other algorithms have the problem of over-segmentation or under-segmentation, with many noise points.

Some evaluation indexes are used in the experiment to measure the segmentation performance of IBKA-OTSU and other algorithms to illustrate the segmentation effect of the IBKA-OTSU more directly. The accuracy (Pre), Matthew’s correlation coefficient (MCC), accuracy rate (acc), dice coefficient (Dice), and Jaccard coefficient (Jac) of different algorithms in remote sensing image segmentation were recorded, respectively. Pre represents the proportion of actual foreground pixels among those marked as foreground. The closer the value is to 1, the fewer pixels with background pixels are mistaken for foreground pixels. The range of Matthews correlation coefficient is [−1, 1], and the larger the value, the better the result. MCC can consider the prediction of foreground and background pixels simultaneously and is a comprehensive evaluation of segmentation images. Accuracy represents the proportion of all correctly predicted pixels to the total pixels, and high accuracy means perfect segmentation. Dice is used to measure the similarity of the image to the fundamental segment after segmentation and is 1 in perfect segmentation. The Jaccard index, also known as the crossover ratio (IoU), is an indicator used to measure the overlap of segmentation results. The Jaccard index is between 0 and 1, and the closer to 1, the result is accurate segmentation. The specific calculation method of the above indicators is shown in Formulas (16)–(20)

(16) $$Pre=\frac{TP}{TP+FP}$$

(17) $$MCC=\frac{TP\times TN-FP\times FN}{\sqrt{\left(TP+FP)\times (TP+FN)\times (TN+FP)\times (TN+FN\right)}}$$

(18) $$acc=\frac{TP+TN}{FN+FP+TP+TN}$$

(19) $$Dice=\frac{2\times TP}{2\times TP+FP+FN}$$

(20) $$Jac=\frac{Dice}{2-Dice}$$

where *TP* is true positive, the measured value is 1, and the true value is 1; *TN* is true negative, the predicted value is 0, and the true value is 0. *FP* is a false positive with a predicted value of 1 and a true value of 0. *FN* is a false negative with a predicted value of 0 and a true value of 1.

From the statistical indicators in Table 4, Table 5, Table 6, Table 7, Table 8 and Table 9, all the indicators of the algorithm proposed in this paper are the best. Pre, MCC, acc, Dice and Jac are all superior to the other five methods. Among them, IBKA-OTSU has the best segmentation results for Image6, with an accuracy of 98.87% and a dice coefficient of 94.68%. The accuracy of the K-means method in the same group of experiments is only 79.84%, and MCC is negative. Although the MCC index of IBKA-OTUS in image 3 and image 5 is not high, 19.66% and 20.60%. The backgrounds of the two images are more complex and harder to separate, resulting in more FP or FN, thereby reducing the MCC. Respectively, it is also significantly better than other methods. In image 5, the MCC index is negative except for the algorithm proposed in this paper and ELM. As can be seen from Table 4, Table 5, Table 6, Table 7, Table 8 and Table 9, compared with IBKA-optimized PCNN remote sensing image segmentation results, the optimized OTSU multi-threshold segmentation method achieves better segmentation results. The segmentation index dice of six random remote sensing images increased by 7.76%, 11.99%, 30.75%, 22.91%, 44.37%, and 18.55%, respectively, indicating that the algorithm proposed in this paper is no worse than the algorithms proposed in recent years in the application of remote sensing images. The improved optimization method proposed in this paper can effectively segment remote-sensing images. The IBKA has more accurate segmentation results and less image distortion than the other five algorithms

## 6. Conclusions

This paper introduces a novel swarm intelligence algorithm called IBKA-OTSU, which integrates multi-threshold image segmentation. Initially, we improved the four key steps of the black-winged kite optimization algorithm. This enhancement significantly increased the algorithm’s convergence speed, optimized its results, and bolstered its robustness by employing the inter-class variance formula as the fitness function. The multi-threshold segmentation process works in conjunction with IBKA, utilizing swarm intelligence techniques for both global and local research to identify the optimal combination of thresholds. This method effectively tackles the challenges of low segmentation efficiency and poor accuracy that are often associated with traditional multi-threshold segmentation approaches. Experimental results demonstrate that the performance of the improved IBKA-OTSU algorithm exceeds that of commonly used classical algorithms. This algorithm has been specifically applied to optimize multi-threshold segmentation in remote-sensing images. The results indicate that this method possesses excellent anti-noise capabilities, produces clear segmented images, and achieves high contrast. Subsequently, further research is considered on the data privacy issues when applied to large-scale data in resource-constrained environments [16].

Future experiments will investigate how different thresholds impact the algorithm’s performance and segmentation results. Given the algorithm’s effectiveness in remote sensing image segmentation, we plan to expand this research into other fields to further validate its optimization performance.

## Figures and Tables

**Table 2:** CEC2019 benchmark functions.

No.	Functions	$F_{i}^{*}=F_{i}(x^{*})$	D	Search Range
1	Storn’s Chebyshev Polynomial Fitting Problem	1	9	[−8192, 8192]
2	Inverse Hilbert Matrix Problem	1	16	[−16,384, 16,384]
3	Lennard-Jones Minimum Energy Cluster	1	18	[−4, 4]
4	Rastrigin’s Function	1	10	[−100, 100]
5	Griewangk’s Function	1	10	[−100, 100]
6	Weierstrass Function	1	10	[−100, 100]
7	Modified Schwefel’s Function	1	10	[−100, 100]
8	Expanded Schaffer’s F6 Function	1	10	[−100, 100]
9	Happy Cat Function	1	10	[−100, 100]
10	Ackley Function	1	10	[−100, 100]

**Table 3:** Performance of different optimization algorithms on CEC2019.

		IBKA	BKA	GWO	WOA	SSA	DBO	SO	SWO
F1	min	**1**	1	1	42.0496	1	1	1	1
	worse	**1**	1	167,908.275	14,123,044.7	1	3,190,589.70	1	48,008.4845
	mean	**1**	1	134.6305	2,353,183.51	1	6587.9074	1	1.2904
F2	best	**4.0371**	4.21157	9.2588	2068.4985	4.2164	4.0526	5	5
	worse	**5**	5	789.9751	17,348.6371	5.0000	4277.5145	5	76.9223
	mean	**4.2546**	4.4769	105.6988	8498.2389	4.2739	65.7948	5	5.3657
F3	best	**1**	1.0004	1.0072	1.0003	1.4095	1.4091	5.6374	4.5387
	worse	**1.4091**	7.5346	7.7026	8.6188	9.7116	8.5662	9.1535	9.1666
	mean	**1.4091**	4.5933	1.7637	4.6166	6.1011	3.6285	7.1404	6.9518
F4	best	**4.9798**	13.9557	8.8933	15.2379	10.9495	8.3915	79.5687	11.1698
	worse	**37.8134**	86.4677	38.5093	124.4226	98.5046	55.7225	152.4794	51.9978
	mean	**16.5389**	49.9627	16.7283	56.4321	40.7415	38.5910	147.2561	35.6282
F5	best	**1.0295**	1.2094	1.05043	1.3183	1.0320	1.0344	50.7073	1.5634
	worse	**1.4254**	18.8652	4.4392	4.2704	1.6053	2.7214	197.9949	3.0960
	mean	**1.1203**	10.4987	1.6904	1.7593	1.1678	1.1279	176.0122	2.1588
F6	best	**1.0246**	4.0724	1.2028	4.8134	1.4760	3.2679	8.7703	3.1140
	worse	6.8977	12.0787	**6.2214**	12.1683	10.5661	9.2392	14.8330	8.0240
	mean	**3.6986**	7.5254	4.7105	7.3367	6.6280	4.2458	13.5858	4.1242
F7	best	**8.0797**	360.7879	243.3382	544.5011	373.5402	268.3831	1559.9227	493.8957
	worse	**1475.5846**	1659.8505	1673.3356	1782.9354	1685.9088	1876.0145	2787.3902	1691.4760
	mean	**684.1900**	795.5225	705.1439	1406.2094	1068.6049	1166.7293	2348.5336	1262.0847
F8	best	**2.5993**	3.1975	2.7658	3.5126	2.7491	3.1786	4.8072	4.0551
	worse	**4.5160**	4.9937	4.5287	5.0846	5.0386	4.9804	5.2852	5.0002
	mean	**3.2315**	4.3818	3.9322	4.2938	4.0016	4.0539	5.2522	4.4134
F9	best	**1.0281**	1.0827	1.0619	1.1769	1.0916	1.0825	3.1420	1.1636
	worse	**1.3093**	3.7570	1.3385	1.7470	1.7388	1.7293	5.4486	1.7068
	mean	**1.1355**	1.1970	1.1429	1.3975	1.4356	1.2752	4.7094	1.3222
F10	best	**1**	5.3302	2.2478	20.9896	2.6462	3.0243	21.3019	11.2906
	worse	21.3808	21.3937	21.5350	21.4902	**21.3607**	21.5185	21.7270	21.5659
	mean	**16.3895**	20.9072	21.2696	21.3423	19.8526	18.9072	21.5257	21.2715

**Table 4:** The experimental results of image 1 using different methods.

Evaluation Metrics	Method					
	IBKA-OTSU	ELM	K-Means	PCNN	OTSU	IBKA-PCNN
Pre	98.78%	94.24%	37.68%	95.81%	96.69%	86.69%
MCC	49.77%	40.07%	−59.10%	46.13%	16.13%	20.28%
acc	68.08%	58.15%	20.45%	64.21%	27.47%	57.50%
Dice	76.51%	66.69%	30.86%	72.62%	14.71%	68.75%
Jac	61.96%	50.03%	18.24%	57.02%	7.94%	52.38%

**Table 5:** The experimental results of image 2 using different methods.

Evaluation Metrics	Method					
	IBKA-OTSU	ELM	K-Means	PCNN	OTSU	IBKA-PCNN
Pre	93.65%	55.47%	83.65%	79.39%	89.57%	72.90%
MCC	89.14%	54.26%	85.20%	39.47%	88.88%	68.93%
acc	94.66%	68.69%	92.01%	69.34%	94.36%	83.30%
Dice	93.49%	71.79%	91.00%	42.51%	93.33%	81.44%
Jac	87.78%	56.00%	83.50%	26.99%	87.51%	68.69%

**Table 6:** The experimental results of image 3 using different methods.

Evaluation Metrics	Method					
	IBKA-OTSU	ELM	K-Means	PCNN	OTSU	IBKA-PCNN
Pre	95.79%	93.55%	71.47%	94.85%	92.27%	67.58%
MCC	19.66%	18.60%	−22.28%	10.25%	−3.32%	−9.17%
acc	73.94%	59.73%	52.53%	47.56%	36.95%	41.38%
Dice	86.82%	72.28%	68.70%	59.16%	44.71%	56.07%
Jac	76.71%	56.59%	52.33%	42.00%	28.80%	38.96%

**Table 7:** The experimental results of image 4 using different methods.

Evaluation Metrics	Method					
	IBKA-OTSU	ELM	K-Means	PCNN	OTSU	IBKA-PCNN
Pre	91.03%	86.94%	15.86%	75.02%	89.28%	70.25%
MCC	58.94%	49.04%	−68.99%	17.19%	7.96%	16.81%
acc	79.14%	72.77%	14.72%	48.11%	41.02%	58.50%
Dice	82.31%	76.16%	14.96%	31.81%	7.67%	63.97%
Jac	69.94%	61.50%	8.09%	18.91%	3.99%	47.03%

**Table 8:** The experimental results of image 5 using different methods.

Evaluation Metrics	Method					
	IBKA-OTSU	ELM	K-Means	PCNN	OTSU	IBKA-PCNN
Pre	96.66%	95.00%	45.64%	66.63%	59.58%	48.36%
MCC	20.60%	17.69%	−31.31%	−1.30%	−1.44%	−21.47%
acc	73.40%	72.51%	25.77%	29.92%	28.58%	35.02%
Dice	87.30%	85.45%	45.39%	16.11%	11.54%	49.74%
Jac	77.47%	74.60%	29.36%	8.76%	6.12%	33.10%

**Table 9:** The experimental results of image 6 using different methods.

Evaluation Metrics	Method					
	IBKA-OTSU	ELM	K-Means	PCNN	OTSU	IBKA-PCNN
Pre	98.87%	96.03%	79.84%	98.22%	98.73%	96.99%
MCC	72.60%	62.51%	−6.63%	31.03%	71.86%	40.12%
acc	90.10%	86.70%	78.21%	52.85%	89.59%	66.47%
Dice	94.68%	92.18%	87.86%	60.95%	94.43%	76.13%
Jac	89.91%	85.50%	78.35%	43.83%	89.45%	61.46%

## Data Availability

No new data were created or analyzed in this study.
